# Surveillance of the ‘bud event of norovirus-associated gastroenteritis’ in schools: does it work in the prevention of norovirus infection outbreaks in Shanghai?

**DOI:** 10.1017/S0950268820000965

**Published:** 2020-05-08

**Authors:** Yi He, Yinhao Lu, Caoyi Xue, Enguo Li, Qinghui Zhang, Fang Xu, Huanyu Wu, Chunyan Luo, Biao Xu

**Affiliations:** 1 Department of Epidemiology, School of Public Health, Fudan University, Shanghai, China; 2 Shanghai Municipal Center for Disease Control and Prevention, Shanghai, China; 3 Pudong District Center for Disease Control and Prevention, Shanghai, China; 4 Jing'an District Center for Disease Control and Prevention, Shanghai, China; 5 Songjiang District Center for Disease Control and Prevention, Shanghai, China; 6 Key Laboratory of Public Health Safety (Fudan University), Ministry of Education, Fudan University, Shanghai 200032, China

**Keywords:** Bud event, Norovirus, Norovirus-associated gastroenteritis, school, Shanghai

## Abstract

Outbreaks of norovirus-associated gastroenteritis have been reported in schools in recent decades in China. For early warning and response to infectious disease outbreaks, the Shanghai Infectious Diseases Bud Event Surveillance System (IDBESS) was established in 2016. Bud event is a term used for the early sign of a potential infectious disease outbreak in public settings when the first few cases appear. This study aimed to describe the epidemiological characteristics of Norovirus-associated gastroenteritis bud events from June 2016 to December 2017 and to understand factors influencing the severity of events. Data were extracted from the IDBESS, supplemented by field investigations and school absence surveillance. In total, 189 bud events of Norovirus-associated gastroenteritis were reported in schools and kindergartens, affecting 3827 individuals and 52.38% happened in primary schools. The attack rate of Norovirus-associated gastroenteritis was 3.82% on average in students in the affected schools. In each event, case numbers varied between 5 and 148, with a median of 16. The duration of bud events lasted for 2 days on average. School absence happened in 47.93% (1797/3749) of affected students and the average duration of absence was 3.07 days. It was found that a longer delay before reporting was associated with a longer-lasting duration of bud event (OR = 2.25, 95% CI: 1.65, 3.07). In conclusion, ascribed to the sensitive threshold for alerting and the timely field investigation, the surveillance of bud events of Norovirus-associated gastroenteritis is effective in the control of Norovirus infection among preschool children and students in Shanghai.

## Introduction

Norovirus (Norovirus), a member of the Caliciviridae family, is a highly contagious virus with a low infection dose of approximately 20 virus particles [1–3]. Norovirus can be divided into 10 genogroups [4], and GII, GI and GIV can cause human infection [5]. The GII genogroup is dominant in several epidemic seasons [6, 7] and has become the leading genogroup of Norovirus outbreaks [2, 8]. The illness of Norovirus infection is characterised by acute onset and an illness duration of 1–3 days and is self-limiting [9]. It causes vomiting, diarrhoea, mild fever and nausea. Diarrhoea is more commonly observed in adults while vomiting is more common in children [5]. The diagnosis of Norovirus infection is mainly based on the detection of the nucleic acid of the virus by RT-PCR. The detection of Norovirus antigens by ELISA has also been applied for diagnosis in China [5].

Norovirus is the leading cause of acute gastroenteritis (AGE) outbreaks. Approximately half of AGE outbreaks reported in the USA and Europe were due to Norovirus [9]. In crowded and semi-closed settings such as schools and kindergartens, Norovirus infection spreads broadly and rapidly, and it can be transmitted via food, water, vomitus, etc[3, 10–14]. Lee *et al*., analysed 121 school AGE outbreaks published between 1998 and 2008 and identified that Norovirus infection accounted for 21.5% of the outbreaks [15]. The general principles to prevent and control Norovirus infection outbreaks were hand hygiene, timely isolation and treatment of patients and environmental disinfection [2].

Outbreaks of Norovirus-associated gastroenteritis in China are most frequently reported in the period of October–December and the following March. Norovirus outbreaks happen more often in southern provinces and in urban areas than in rural areas [11]. The national surveillance system on Norovirus-associated gastroenteritis was initiated relatively late and the reporting sensitivity and timeliness were to be improved [16]. In Shanghai, it is also common to have Norovirus outbreaks each year among students, as reported [17–19]. The Public Health Emergency Event Surveillance System (PHEESS) is a reporting system established by the national CDC for monitoring disease outbreak. PHEESS has set up a standardised threshold for reporting in terms of a number of cases. However, the standardised threshold was not sensitive enough for Shanghai considering its huge population size and, high population density. In order to give an early alert and implement a field investigation before the PHEESS threshold is triggered, varied thresholds was set up in Shanghai before 2016 based on the nature of events, the severity of diseases and the affected locations. The case threshold for alerting in districts with larger areas and higher population density was less sensitive than that in districts with smaller areas and lower population density. To provide early warning and to respond to major infectious diseases, the Shanghai Municipal Centre for Disease Control and Prevention (Shanghai CDC) initiated the Shanghai Infectious Diseases Bud Event Surveillance System (IDBESS) in June 2016. Bud event is a term used for the early sign of a potential infectious disease outbreak in public settings when the first few cases appear. Following the national reporting criteria for public health emergencies, the threshold for bud events of major infectious diseases has been set up based on the number of cases, days of events and type of settings. Once a bud event threshold is reached, an alert will be triggered and the corresponding district should respond in a timely manner and implement field investigations as requested [20].

In addition to the IDBESS, Shanghai has established a school absence surveillance system (SASS) since 2010 to monitor student absence and disease outbreaks. The SASS is an online system covering all kindergartens, primary schools, middle schools, high schools and other schools. School health personnel collect and report the student and staff absences every school day and the public health professionals in municipal and district CDCs are responsible for analysis and the appropriate response.

This study aimed to describe the epidemiological characteristics of the bud events of Norovirus-associated gastroenteritis and to understand factors influencing the spread and severity of the events based on data from the IDBESS, field investigations and SASS.

## Subjects and methods

### Subjects

The study subjects were preschool children and students in each Norovirus-associated gastroenteritis bud event reported from kindergartens, primary schools and middle schools in Shanghai from 1 June 2016 to 31 December 2017. Information on affected individuals was extracted from the IDBESS, supplemented by the case-based field investigation and school absence surveillance system (the SASS).

### Methods

A Norovirus-associated gastroenteritis bud event is defined by the following three criteria: (1) case count: ⩾six affected individuals in 1 day or ⩾10 affected individuals in 3 days within the same class or dormitory or ⩾five affected individuals in 1 week in the same school or kindergarten; (2) clinical manifestation: diarrhoea ⩾3 times per day or vomiting ⩾2 times per day; and (3) at least one case confirmed as Norovirus infection by RT-PCR nucleic acid detection by the reference laboratory in the district CDC.

The IDBESS information included the name and district of the school or kindergarten, the date of the report of the bud event, the onset date of the first case, the number of affected individuals (students and staff) and classes involved, the number of hospital visits and laboratory confirmations, the number of students in the corresponding class or dormitory, the date of the last case identified, the date of bud event closure, etc.

Once a bud event of Norovirus-associated gastroenteritis is reported, case investigation by the district CDCs should be carried out immediately using a structured questionnaire. The questionnaire covers information on the sex, class and school of the affected individual, the date of disease onset and the dates of the clinic visit and school absence. Based on the investigation, the route of transmission is constructed and determined according to the *Guidelines on Outbreak Investigation, Prevention and Control of Norovirus Infection (2015)* [5].

The SASS set in the municipal CDC receives daily online reporting from health personnel in each school and kindergarten in Shanghai for the details on school absences, which includes the sex, class and grade of the affected individual and the date and main reason for absence.

### Statistical analysis

The non-ID data were exported from the SQL database and cleaned. Stata/SE 14 (Stata Corp LLC, USA) was used for statistical analysis. An ANOVA or rank-sum test was applied for continuous data and the chi-squared test or Fisher test was applied for discrete data in the univariate analysis. Logistic regression was used for multi-variate analysis to calculate odds ratios (ORs) and 95% confidence intervals (CIs) at a significance level of *P* < 0.05.

The season was classified as spring (March–May), summer (June–August), autumn (September–November) and winter (December–the following February).

The attack rate in students was defined as the number of affected students divided by the total number of students at risk in the affected schools or kindergartens.

According to the literature [21], the average incubation period is 1.2 ± 1.64 days. Thus, taking 3 days as the longest incubation period, the duration of bud events in this study was grouped into 0–2 days, 3–5 days, 6–8 days and ⩾9 days.

## Results

### Epidemiological characteristics of Norovirus-associated gastroenteritis bud events

In total, 215 Norovirus-associated gastroenteritis bud events were reported from June 2016 to December 2017 in Shanghai; of these, 189 (87.91%) happened in schools and kindergartens. Primary schools accounted for 52.38% of the events, followed by kindergartens (39.15%). There were 3840 affected individuals reported from the 189 events; of them, questionnaire investigations were completed for 3827, while 13 cases from two bud events were missing. Of the investigated affected individuals, 3749 were students and 78 were the staff. School absence data were matched in 1797 students. The average attack rate of Norovirus-associated gastroenteritis among students in bud events was 3.82% and the highest attack rate was found in kindergartens (5.86%). The attack rates varied among different types of schools with statistical significance (*P* < 0.001) (Table 1). The case number per bud event ranged from 5 to 148, with a median of16 (IQR 12–23).

**Table 1.** Major indicators for NoVs-associated gastroenteritis bud events in different types of schools

Major indicators		Kindergarten	Primary school	Middle school and high school	Total
No. of bud events		74	99	16	189
No. of cases	Students	1119	2290	340	3749
	Staff	28	30	20	78
	Total	1147	2320	360	3827
Attack rate (%)		5.86	2.47	2.72	3.82
No. of hospital visit		714	1150	172	2036
No. of lab confirmed cases		316	462	67	845
No. of school absence		532	1117	148	1797
School absence duration (days)	Mean	3.4	2.92	2.57	3.07
	SE	0.34	0.22	0.34	0.18

Genogroup information was available for 124 events, including 122 (98.39%) under GII and 2 (1.61%) under GI. Human-to-human transmission accounted for 90.48% (171/189), followed by foodborne transmission (2.65%, 5/189) and both (6.88%, 13/189). No waterborne transmission was observed.

**Fig. 1.**
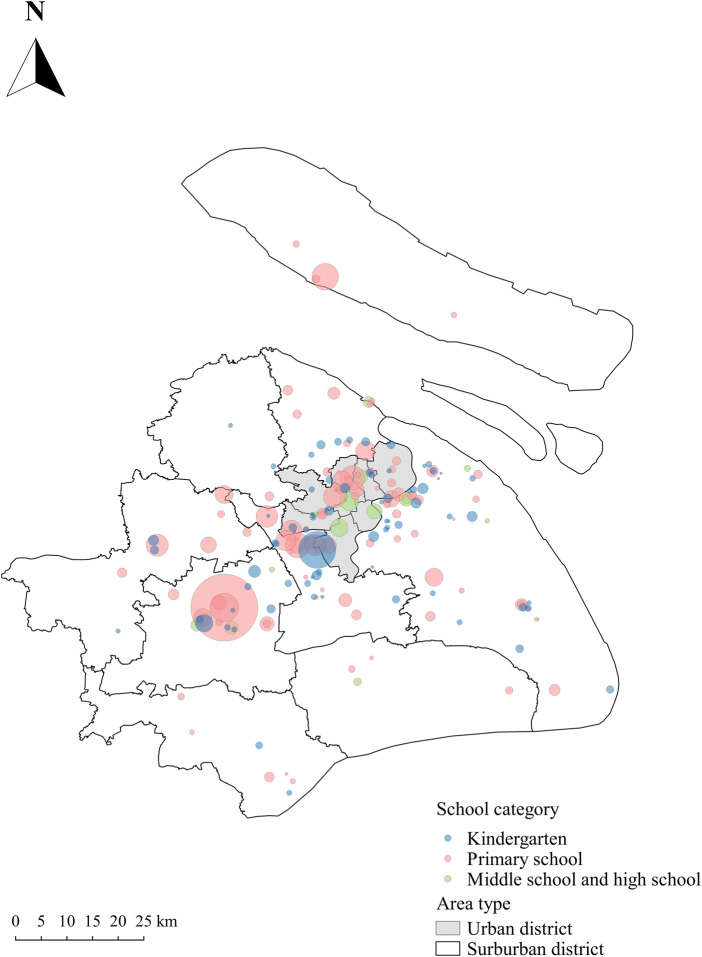
Geographic distribution of NoVs-associated gastroenteritis bud events in Shanghai, China.

Reports of Norovirus-associated gastroenteritis bud events came from all 16 districts of Shanghai and the number of reports ranged from 1 to 55, with a median of seven events. A total of 149 bud events were located in suburban areas (78.84%) and 40 were located in urban areas (21.16%). The geographical distribution and size of the reported Norovirus-associated gastroenteritis bud events are shown in Figure 1.

**Fig. 2.**
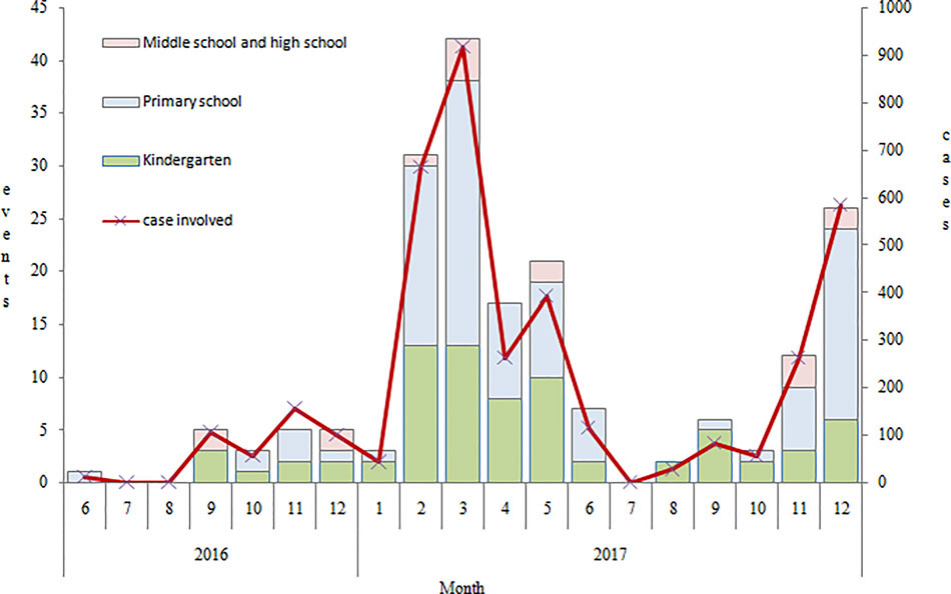
Time distribution of NoVs-associated gastroenteritis bud events in Shanghai from June 2016 to December 2017.

As indicated in Figure 2, the Norovirus-associated gastroenteritis bud events presented seasonal variation, with the lowest during the school summer break period and peaks in winter and spring, although the study period was not long enough to establish seasonality.

The duration of the bud events lasting from the onset of the first case to the last case varied between 0 (within 1 day) and 28 days, with a median of 2 days. Significant differences in duration were observed among different types of schools (*P* < 0.001) and the shortest was in kindergartens (1 day in median) (Table 2).The median time intervals from the onset of the first case to bud event reporting and to bud event closure were 2 days (range 0–11 days) and 7 days (range 0–28 days), respectively. There were no significant differences in these two durations among different types of schools (*P* > 0.05).

**Table 2.** The time intervals of NoVs-associated gastroenteritis bud events among different school types in Shanghai

The time intervals		Kindergarten	Primary school	Middle school and high school	Total
First case to Last case^a^	Median	1	2	2	2
	IQR^b^	(0,2)	(1,6)	(1,3.5)	(0,4)
First case to reporting^c^	Median	1	2	2	2
	IQR^b^	(1,3)	(1,3)	(1,2.5)	(1,3)
First case to stop response^d^	Median	6	8	9	7
	IQR^b^	(5,9)	(5,12)	(6.5,11)	(5,11)

a Represents the time interval from the onset of the first case to the last case.

b IQR: Interquartile range.

c Represents the time interval from the onset of the first case to the reporting of the Novs-associated gastroenteritis bud event.

d Represents the time interval from the onset of the first case to the Novs-associated gastroenteritis bud event close.

### Epidemiological characteristics of Norovirus-associated gastroenteritis bud events

Of the 3827 affected individuals from the 189 Norovirus-associated gastroenteritis bud events, 2121 were boys and 1706 were girls. The sex ratio (M/F) varied from 0.18 to 9, on average, 1.2, with a significant difference among different types of schools. Due to the infection, 2036 affected individuals (53.20%) visited hospitals and 845 (22.08%) received laboratory confirmation of Norovirus infection; no deaths were reported. Affected individuals from primary schools accounted for 60.62% of cases.

The frequency of school absence was 47.93%, i.e. 1797 preschool children and students among the 3749 affected individuals and the length of school absence was 3.07 days on average. There were no significant differences among different types of schools for either the frequency or the duration of school absence (*P* > 0.05).

### The associations between cases and public health factors and the duration of Norovirus-associated gastroenteritis bud events

After grouping the duration of Norovirus-associated gastroenteritis bud events into 0–2, 3–5, 6–8 and ⩾9 days, it was found that the sex ratio of affected individuals, the location of schools (urban/suburban), the type of school, the number of classes affected and the time interval between the onset of the first case and the initiation of bud event reporting were significantly associated with the duration of the event in the univariate analysis. Multi-variate analysis using logistic regression was performed with indicators of the affected individuals, hospital visits, school absences, type of school, location of the school, seasons and time to reporting as independent variables. The time interval between the onset of the first case and the reporting of bud events was significantly associated with the duration of the events, with an OR equal to 2.25 (95% CI: 1.65, 3.07) and the more classes affected, the longer the events lasted (OR = 1.56, 95% CI: 1.34, 1.81) (Table 3).

**Table 3.** Analysis for the association between cases and public health factors and the duration of NoVs-associated gastroenteritis bud events

Factors	Univariate			Multivariate		
	OR	*P*	95%CI	OR	*P*	95%CI
Sex ratio of students	1.35	0.029	(1.03,1.77)	1.24	0.164	(0.91,1.69)
Location						
Urban	1.00	–	–	1.00	–	–
Suburban	2.89	0.017	(1.21,6.92)	2.29	0.194	(0.66,7.99)
Type of school						
Kindergarten	1.00	–	–	1.00	–	–
Primary school	3.65	<0.001	(1.82,7.32)	1.31	0.620	(0.45,3.77)
Middle and high school	2.60	0.098	(0.84,8.09)	1.92	0.419	(0.39,9.36)
1st case to event report^a^	1.79	<0.001	(1.47,2.18)	2.25	<0.001	(1.65,3.07)
Classes affected	1.39	<0.001	(1.27,1.52)	1.56	<0.001	(1.34,1.81)
Absence proportion	0.55	0.336	(0.17,1.84)	1.50	0.624	(0.30,7.58)
Absence days	1.15	0.095	(0.98,1.34)	1.17	0.129	(0.96,1.43)
Seasons						
Spring	1.00	–	–	1.00	–	–
Summer	0.51	0.408	(0.10,2.52)	0.03	0.042	(0.001,0.87)
Autumn	1.53	0.302	(0.68,3.42)	1.11	0.862	(0.33,3.78)
Winter	1.28	0.484	(0.64,2.53)	1.09	0.864	(0.40,2.96)

a Represents the time interval from the onset of the first case to the reporting of the Novs-associated gastroenteritis bud event.

## Discussion

Norovirus-associated gastroenteritis is called ‘winter vomiting disease’ [22] and Norovirus outbreaks have obvious seasonality with the winter peak (December to the following February) [17, 23–25]. In this study, the Norovirus-associated gastroenteritis bud events also presented seasonal variation, with peaks in winter and spring. This coincided with some countries' seasonal peak delay in the literature [26] and was also consistent with the Chinese Norovirus outbreak time distribution [11, 27, 28].

Primary schools accounted for more than half of the Norovirus-associated gastroenteritis bud events and affected individuals, which was consistent with other reported studies in Shanghai [28]. However, Norovirus gastroenteritis outbreaks were more frequently reported in middle schools and high schools than in primary schools in China [11, 27].

For the transmission route, human-to-human transmission accounted for 90.48% in Shanghai. This was mainly due to improper disinfection measures for the vomitus in class and the lack of timely isolation of index cases. It was consistent with the result of CaliciNet China [14]. The proportion of human-to-human transmission was much higher than that reported by the NORS in the USA [29, 30]. Foodborne transmission accounted for only 2.65%. Even taking 6.88% dual foodborne and human-to-human transmission routes into consideration, the percentage was still lower than the 16% reported in the literature [8]. All five Norovirus-associated gastroenteritis bud events with foodborne transmission were attributed to food handler infection, which in turn contaminated food and caused further spread; this observation was also seen in the studies done by the CDC of the USA [22, 31]. In this study, no waterborne transmission was observed, although it was reported in the literature [29, 32].

In terms of aetiology, 98.39% of the genogroup GII Norovirus-associated gastroenteritis bud events occurred from June 2016 to 2017, which was consistent with the results of paediatric diarrhoea research in Shanghai and Hangzhou [7]; moreover, it was also consistent with the Japanese findings in outbreaks of kindergartens [33]. However, this percentage was higher than the reported GII proportion in some districts of Shanghai [17] and was also higher than the genogroup GII proportion (72.73%) of Norovirus-associated gastroenteritis outbreaks in China [11].

The median number of cases per bud event in Shanghai was 16 (IQR 12–23), which was lower than the 20 (IQR 10–35) reported by Germany [34]. The average student attack rate was 3.82% in this study and the highest attack rate for kindergartens was 5.86%, which was much lower than the literature-reported attack rate of over 50% in hospitals and nursing homes [35]. It was reported that the primary attack rate in school settings during Norovirus outbreaks was 28%, while in this study, the average attack rate among Norovirus-associated gastroenteritis bud events with a duration less than 2 days (regarded as a point exposure) was only 2.8% and the highest was 22.3%, which were much lower than the value reported by other countries in the literature [8]. Moreover, the average attack rate in Shanghai was lower than that in Beijing (11.03%) [10].

The lower median number of cases per bud event and the lower attack rates found in our study compared with other studies reported in the literature are probably attributable to two main reasons. First, our values were associated with timely reporting. The average time interval between the first case onset and the reporting of Norovirus-associated gastroenteritis bud events was only 2 days. Because peak Norovirus shedding occurs 2–5 days after infection and is mainly transmitted via faeces and vomitus [2], the sensitivity threshold of the IDBESS for alerting and the early response by the community and district CDC based on the IDBESS and the SASS decreased the attack rate and shortened the duration of bud events. Second, our lower values were also related to the sensitive reporting and response threshold for the Norovirus-associated gastroenteritis bud events in Shanghai. According to the *Guidelines on Outbreak Investigation, Prevention and Control of Norovirus Infection (2015)* [5], the national threshold for Norovirus outbreaks requires at least two laboratory-confirmed cases, while in Shanghai, the threshold for a Norovirus-associated gastroenteritis bud event asks for only one laboratory-confirmed case.

This study revealed that bud events that were reported later were associated with a longer duration of the Norovirus-associated gastroenteritis bud event (OR = 2.25, 95% CI: 1.65, 3.07). The average and maximum durations of the Norovirus-associated gastroenteritis bud events were 2 days and 6 days, respectively, which were much lower than the 19 days for hospitals and the 16 days for nursing homes reported by other countries[35] and lower than the 8–10 days reported in China[11, 27]. In addition to the two reasons discussed above, the low attack rate was probably associated with a high proportion of student hospital visits. In this study, approximately 50% of affected individuals sought medical care in hospitals and this proportion was much higher than the 10% reported in the USA [9]. In a short period of time, a large number of students seeking medical treatment can easily arouse the vigilance of hospitals, which makes earlier reporting possible. Therefore, it is crucial to educate students to seek medical care in a timely manner, which will in turn play an important role in early reporting by hospitals and decrease further spread.

There were some limitations in this study. First, the majority of the positive samples were not further tested for gene sequence analysis; therefore, it was not possible to further analyse the impact of genogroups on attack rates and durations, as seen in the literature on US outbreaks [36]. Second, it was reported in the literature that hospitals, long-term care facilities (LTCFs), restaurants and cruise ships were the main settings for Norovirus outbreaks [9, 13, 37] and only 3.8% of outbreaks were in school settings [31]. Outbreaks in hospitals were reported to be associated with higher hospitalisation and mortality rates [38]. However, schools and kindergartens accounted for 87.91% of the Norovirus-associated gastroenteritis bud events in this study. This finding was probably related to inadequate identification capability for other collective units; thus, this study covered only schools and kindergartens.

It is imperative for the IDBESS to combine epidemiological data and molecular information in future to understand the pathogenic impact on the bud events. Furthermore, the IDBESS could be applied to other regions in China for early alert and response of Norovirus outbreaks.

## Conclusion

Norovirus-associated gastroenteritis outbreaks in schools are relatively small in size and short in length. The average attack rate in students was 2.47–5.86%. Ascribed to the sensitive threshold for alerting and the timely field investigation, the surveillance of Norovirus-associated gastroenteritis bud events is effective in the control of Norovirus infection among preschool children and students in Shanghai.
